# The Association of Vigorous Activity With Sleep Patterns in the Adventist Health Study 2 Authors

**DOI:** 10.1177/15598276261471058

**Published:** 2026-07-26

**Authors:** Michael Paalani, Daijah Lofton, Salim Serrano, Jones Ampadu Adjei, Alaa Alabadi-Bierman, Jerry W. Lee, W. Lawrence Beeson, Hildemar Dos Santos

**Affiliations:** 1 Preventive Care DrPH Program, 48766 Loma Linda University School of Public Health, Loma Linda, CA, USA (MP, DL, SS, JAA, AA-B, JWL, WLB, HDS)

**Keywords:** Adventist Health Study 2, AHS-2, physical activity, sleep, sleep patterns, insomnia

## Abstract

Physical activity may promote sleep, but few studies examined whether vigorous exercise is associated with difficulty falling asleep or waking in the middle of the night. This study examined the relationship between these variables. The Adventist Health Study-2 (AHS-2) questionnaireline was completed by approximately 97000 churchgoers in North America to investigate relationships between lifestyle, including exercise habits, and chronic disease. A randomly selected subset participated in the Biopsychosocial Religion and Health Study (BRHS), including about 11 000 respondents who answered questions about psychosocial and religious issues, including sleep patterns. After excluding participants with chronic disease, 3960 remained: 2502 women (1502 Whites and 1000 Blacks) and 1458 men (1038 Whites and 420 Blacks). Logistic regressions were adjusted for age, gender, race, body mass index (BMI), diet, education, alcohol, caffeinated-beverage use, hours of sleep, and napping. Increased frequency of vigorous physical activity was protective against difficulty falling asleep (OR = 0.97; 95% CI = 0.94-0.98; *P* = 0.03). Higher odds of difficulty falling asleep were observed among female participants, increasing age, weekday napping, and sleeping 9 hours or more per night. Vigorous physical activity was associated with reduced difficulty falling asleepline but showed no relation to waking during the night.

“We found that the frequency of vigorous activity might have been protective against difficulty falling asleep in a multiracial population.”

## Introduction

Abnormal sleep patterns constitute an ever-increasing public health hazard.^1^ On average, over 35% of the U. S. population gets by on less than seven hours of sleep per night.^2^ According to Blackwelder, Hoskins, and Huber,^3^ insufficient sleep affects over 18% of the adult population. The National Institutes of Health indicated that over 10% of Americans are suffering from severe sleep deprivation. Sleep latency—the amount of time spent trying to sleep and actually falling asleep—is rapidly increasing, and this has been linked to lifestyle choices that lead to anxiety and stress, and lack of physical activity.^4^ Lack of sleep may lead to mood disorders like low self-esteem and anxiety^5^ and weight gain.^6^ Other sleep disturbances, including waking in the middle of the night and being unable to go back to sleep after early awakening, have not been studied extensively in relation to exercise.

One of the modalities suggested for improving sleep length and quality is moderate physical activity.^7^ Activity increases the metabolic state and central nervous system temperature and thus may promote a more favorable sleep architecture.^8^ Lack of physical activity may lead to an increased likelihood of frequent awakening during the night,^9^ sleeping less deeply,^10^ and taking longer to fall asleep.^11^ According to the National Center for Chronic Disease Prevention and Health Promotion (Division of Nutrition, Physical Activity and Obesity), performing daily aerobic physical activities for 30 to 60 minutes on three to five days a week may improve sleep.^12^

Studies regarding exercise and sleep have shown that regular activity, or as little as walking the equivalent of over six blocks at a normal pace on a daily basis, was associated with reduced risk of sleep disorders, regardless of gender.^13^ However, other studies have indicated that more exercise is better. Tworoger and colleagues in 2003, performed an intervention study suggesting that a weekly amount of at least 225 minutes of morning physical activity was associated with less trouble falling asleep when compared with less than 180 minutes per week.^14^

Few studies have looked at exercise intensity, though prolonged and vigorous exercise may impair sleep, especially if undertaken close to bedtime.^13^ Vigorous activity is shown to protect against cardiovascular disease^15^ and could be useful in protecting against other health problems, including sleep patterns.^16^ Studies of the association between vigorous activity and sleep patterns need to take into consideration several confounding factors. In addition to sociodemographic factors, lifestyle factors, including diet, napping patterns, and smoking habits, may confound the association. Ingestion of certain beverages may be associated with poor quality of sleep. Caffeine has been shown to disrupt sleep architecture.^17^ Adults ingesting five or more alcoholic drinks a day were more likely to sleep six hours or less than their counterparts with more moderate drinking habits.^18^ Daytime napping could also affect nighttime sleep and may be a marker of inadequate sleep.^19^

In the current study, we examined the association between vigorous activity and sleep patterns in a nonsmoking population, in which we attempted to control for sociodemographic variables as well as potential lifestyle confounders, including weekend and weekday napping, diet, caffeine, and alcohol use. We set out to examine any association between the usual reported frequency of vigorous activity (at baseline) and nocturnal sleep patterns (at follow-up) after adjusting for control variables.

## Methods

The Adventist Health Study (AHS-2) was initiated in 2002 with the goal of investigating the association between diet and cancer risk.^20^ Approximately 97000 church members of the Seventh-day Adventist Church (SDA) aged 30 years or more and able to read and speak English comprised the study population,^21^ completing a detailed questionnaire which was divided into seven main sections: medical history, dietary habits, physical activity, female-health history, census-related and lifestyle-related information, foods with nutrition label consumption pattern, and demographic information.

The first wave of the Biopsychosocial Religion and Health Study (BRHS) comprises a subset of 10988 respondents from the AHS-2 participants who, one to four years after returning the baseline AHS-2 questionnaire, also completed a 20-page questionnaire regarding religious, psychological, social, stress, and health practices.^22^ The BRHS questionnaire included questions about the religious and social environment, health, feelings, religious/spiritual life, relationships, life stress, and medical history.

At AHS-2 baseline, the participants level of vigorous activity was assessed with the question, “How many times per week do you usually engage in regular vigorous activities, such as brisk walking, jogging, bicycling, etc., long enough or with enough intensity to work up a sweat, get your heart thumping, or get out of breath?” Responses were coded on a four-level ordinal scale: never to less than once per week, 1-3 times per week, 4-5 times per week, and 6 or more times per week. Closely related vigorous-activity questions have demonstrated validity and test-retest reliability in Seventh-day Adventist populations,^23^ although the AHS-2 activity question was not specifically revalidated in the present study.

At BRHS follow-up, participants reported how often during the previous four weeks they experienced (1) trouble falling asleep, (2) waking in the middle of the night and finding it difficult to return to sleep, and (3) waking very early and being unable to return to sleep. The response options were rarely or never, sometimes, often, and almost every day. For logistic regression, rarely or never was coded as 0, whereas sometimes, often, or almost every day were combined and coded as 1. At AHS-2 baseline, usual sleep duration was assessed separately by asking, “How many hours do you usually sleep each night?” and was categorized as less than 6, 6-8, or 9 or more hours per night. AHS-2 sleep duration as well as BRHS sleep-symptoms were assessed based on self-report and were not validated against objective sleep measurements in this study.

Baseline AHS-2 covariates included age in years, race (White vs Black), gender (male vs female); BMI in kg/m2 (calculated from self-reported height and weight), dietary pattern (categorized as omnivore vs vegan/lacto-ovo vegetarian), educational attainment (categorized as high school/trade school or less, some college/associate or bachelor’s degree, or master’s degree or higher), alcohol use (categorized as none vs any use), weekday napping (categorized as none, less than 20 minutes, or 20 minutes or more), and weekend napping (categorized as none, less than 40 minutes, or 40 minutes or more). Caffeinated-beverage use (categorized as none vs any use) was obtained from the BRHS follow-up questionnaire. The interval between questionnaires was calculated in years from the AHS-2 and BRHS completion dates.

For the current prospective observational study, subjects who responded to AHS-2 comprised the baseline population if they also participated in the BRHS. In particular, vigorous activity was assessed at baseline, while sleep variables were measured at follow-up, which was an average of three years after the 1-4 year study inclusion range. Respondents were excluded if they had experienced any of the following: stroke lasting at least 24 hours, small stroke lasting less than 24 hours, angina pectoris, rheumatoid arthritis, degenerative arthritis and disk disorders, sciatica/arthritis of the back, asthma, fibromyalgia, or sleep apnea. The following were also excluded: non-church members, those less than 30 years old, and those defined as morbidly obese (BMI >40 kg/m^2^). From an original respondent population of 10 988, these exclusions reduced our study population to 4934. Our final *N* was 4,586, after accounting for missing data.

### Statistical Analysis

A G*POWER analysis was done with *F* as the test family and multiple regression testing *R*^*2*^ deviation from zero.^24^ Sample size, given a .05 alpha, 0.8 power, small effect size (*f*^*2*^ = .02), and 13 as the number of predictors, yielded a minimum respondent sample size of 904.

Using SPSS version 20 (Statistical Package for the Social Sciences, Armonk, New York), we used logistic regression with frequency of vigorous activity taken from the AHS-2 responses as the independent variable, and sleeping patterns taken from the BRHS responses (trouble falling asleep, waking in the middle of the night, waking early and being unable to get back to sleep) as the dependent variables. The final model was adjusted for age, BMI, diet, education, alcohol and caffeinated-beverage use, gender, and race, weekend napping, weekday napping, interval in years between questionnaires, and hours of sleep.

## Results

As shown in Table 1, more Whites than Blacks and more females than males participated in the study. Female respondents were significantly younger than males, and Black respondents were significantly younger than their White counterparts. Blacks had a significantly higher BMI than Whites, with Black females having a significantly higher BMI than any other racial and gender group. While White males had a greater BMI than their female counterparts, the opposite pattern was shown among Blacks (data not shown). The time between questionnaires was approximately three years, and significantly longer in Whites than in Black respondents.

**Table 1.** Respondents’ Age, Education, Lifestyle and Sleep Characteristics by Race and Gender

​	Black		White		*P*
	Female (n = 1000) (%)	Male (n = 420) (%)	Female (n = 1502) (%)	Male (n = 1038) (%)	
Age	​	​	​	​	**<.001**
30-50	44.5	35.7	31.3	26.6	​
51-65	40.0	44.5	39.6	39.2	​
66 and above	15.5	19.8	29.1	34.2	​
Education	​	​	​	​	**<.001**
High/trade school or less	15.5	19.3	20.6	17.3	​
Some college/AS/bachelors	59.7	53.3	66.3	47.8	​
Master’s degree or higher	24.8	27.4	13.1	34.9	​
TV watching	​	​	​	​	**<.001**
Less than 2 hrs.	36.5	39.2	57.3	56.8	​
2 hrs. or more	63.5	60.8	42.7	43.2	​
Exercise frequency	​	​	​	​	**.002**
Never to <1/week	29.5	25.9	28.6	25.9	​
1-3X/week	44.4	44.5	40.5	42.6	​
4-5X/week	22.2	22.9	23.2	22.1	​
≥6/week	3.9	6.7	7.6	9.4	​
Weekend nap	​	​	​	​	.072
<40 minutes	15.3	14.0	18	16.4	​
≥40 minutes	61.4	67.9	48.0	59.7	​
None	23.3	18.1	34.0	23.9	​
Weekday nap	​	​	​	​	**<.001**
None	56.4	44.5	71.4	62.4	​
<20 minutes	20.6	21.4	16.7	20.5	​
≥20 minutes	23.0	34.1	11.9	17.1	​
Diet	​	​	​	​	.084
Vegan/Lacto-ovo veg^a^	28.9	29.5	56.0	52.3	​
Omnivore	71.1	70.5	44.0	47.7	​
Caffeine use	​	​	​	​	**<.001**
No	77.7	74.1	58.1	56.1	​
Yes	22.3	26.0	41.9	43.9	​
Alcohol use	​	​	​	​	.372
No	94.1	92.4	91.9	89.5	​
Yes	5.9	7.7	8.1	10.5	​
Sleeping hours	​	​	​	​	**<.001**
Less than 6 hrs	15.7	21.4	5.0	4.4	​
6-8 hrs	79.0	76.2	88.2	89.6	​
9 hrs or more	5.3	2.4	6.9	6.0	​
Trouble falling asleep	​	​	​	​	**<.001**
Rarely or never	63.9	76.0	55.5	71.4	​
Sometimes/often/everyday	36.1	24.1	44.5	28.6	​
Waking in middle of night	​	​	​	​	**<.001**
Rarely or never	55.3	64.8	45.2	49.8	​
Sometimes/often/everyday	44.7	35.2	54.8	50.2	​
Waking early	​	​	​	​	**<.001**
Rarely or never	59.1	68.1	53.5	51.0	​
Sometimes/often/everyday	40.9	31.9	46.5	49.0	​

^a^Lacto-ovo vegetarian refers to a diet that excludes meat and fish but includes dairy products and eggs.

As seen in Table 1, most respondents had some college education. In general, more females than males reported abnormal sleeping patterns. Also, within each race, more females than males were vegetarians and used caffeinated-beverages.

We found that increasing vigorous activity frequency significantly reduces the reporting of trouble falling asleep (OR = 0.97; 95% CI: 0.94 - 0.98, *P* = .03 (Table 2). However, we did not find any significant association between vigorous activity frequency and waking in the middle of the night (OR = 1.00; 95% CI: 0.97 - 1.03, *P* = .824 (Table 3) nor with waking early and not going back to sleep (OR = 1.02; 95% CI: 0.99 - 1.06, *P* = .141 (Table 4].

**Table 2.** Logistic Regression Relating Vigorous-Activity Frequency to Trouble Falling Asleep, Adjusted for Control Variables (N = 3960).

Effect	Odds Ratio	95% Cls^a^	*P*
Vigorous-activity frequency	0.97	(0.94, 0.98)	.034
Age	1.01	(1.00, 1.02)	**.003**
BMI (Kg/m^2^)	1.01	(0.99, 1.03)	.263
Diet			
Omnivore	Ref.	​	​
Vegan/lacto-ovo veg^b^	0.96	(0.82, 1.11)	.558
Education			
Less than high school	Ref.	​	​
Some college/AS/bachelors	0.80	(0.64, 0.99)	.044
Master’s degree or higher	0.85	(0.71, 1.01)	.065
Alcohol			
No	Ref.	​	​
Yes	1.05	(0.81, 1.34)	.727
Caffeinated-beverage use			
No	Ref.	​	​
Yes	0.75	(0.65, 0.88)	**<.001**
Gender/race			
White male	Ref.	​	​
Black female	1.33	(1.08, 1.64)	**.007**
Black male	0.62	(0.47, 0.82)	**.001**
White female	2.15	(1.80, 2.57)	**<.001**
Weekend napping			
None	Ref.	​	​
<40 minutes	0.94	(0.76, 1.18)	.602
≥40 minutes	1.08	(0.91, 1.28)	.410
Weekday napping			
None	Ref.	​	​
<20 minutes	1.32	(1.10, 1.59)	**.003**
≥20 minutes	1.41	(1.15, 1.71)	**.001**
Interval between AHS-2 and BRHS^c^	0.97	(0.91, 1.03)	.343
AHS-2 hours of sleeping			
6-8 hrs	Ref.	​	​
Less than 6 hrs	0.64	(0.47, 0.86)	**.004**
9 hrs. or more	2.81	(2.22, 3.54)	**<.001**

*Note.* The dependent variable, *trouble falling asleep*, was coded as 1 = yes (reported difficulty falling asleep) and 0 = no (no reported difficulty falling asleep).

^a^CI = confidence intervals.

^b^Lacto-ovo vegetarian refers to a diet that excludes meat and fish but includes dairy products and eggs.

^c^Interval between AHS-2 and BRHS refers to the number of years between completion of the Adventist Health Study-2 (AHS-2) baseline questionnaire and the Biopsychosocial Religion and Health Study (BRHS) follow-up questionnaire.

**Table 3.** Logistic Regression Relating Vigorous-Activity Frequency to Waking in the Middle of the Night, Adjusted for Control Variables (N = 3960).

Effect	Odds RATIO	95% Cl^a^	*P*
Vigorous-activity frequency	1.00	(0.97, 1.03)	.824
Age	1.02	(1.01, 1.02)	**<.001**
BMI (Kg/m^2^)	1.00	(0.98, 1.01)	.594
Diet			
Omnivore	Ref	​	​
Vegan/lacto-ovo veg^b^	1.08	(0.93, 1.25)	.300
Education	​	​	​
Less than high school	Ref.	​	​
Some college/AS/bachelors	0.90	(0.72, 1.09)	.265
Master’s degree or higher	1.00	(0.84, 1.19)	.990
Alcohol			
No	Ref.	​	​
Yes	0.80	(0.62, 1.03)	.077
Caffeinated-beverage use			
No	Ref.	​	​
Yes	0.88	(0.76, 1.03)	.101
Gender/race			
White male	Ref.	​	​
Black female	0.82	(0.68, 0.99)	**.046**
Black male	0.47	(0.37, 0.61)	**<.001**
White female	1.26	(1.06, 1.48)	**.007**
Weekend napping			
None	Ref.	​	​
<40 minutes	1.24	(1.01, 1.53)	**.041**
≥40 minutes	1.19	(0.01, 1.40)	**.039**
Weekday napping (none is the referent category)			
None	Ref.	​	​
<20 minutes	1.33	(1.12, 1.59)	**.002**
≥20 minutes	1.22	(1.01, 1.47)	**.043**
Interval between AHS-2 and BRHS^c^	0.98	(0.92, 1.04)	.489
AHS-2 - hours of sleeping			
6-8 hrs	Ref.	​	​
Less than 6 hrs	0.86.	(0.65, 1.14)	.289
9 hrs. or more	2.65	(2.09, 3.37)	**<.001**

*Note.* The dependent variable, *waking in the middle of the night*, was coded as 1 = yes (reported waking during the night) and 0 = no (did not report waking during the night).

Exercise frequency was modeled as a continuous variable in the original published analysis. Exercise was coded on a four-level ordinal scale (never to <1 time/week, 1-3 times/week, 4-5 times/week, ≥6 times/week), and odds ratios represent the change in odds associated with a one-category increase in exercise frequency.

^a^CI = confidence intervals.

^b^Lacto-ovo vegetarian refers to a diet that excludes meat and fish but includes dairy products and eggs.

^c^Interval between AHS-2 and BRHS refers to the number of years between completion of the Adventist Health Study-2 (AHS-2) baseline questionnaire and the Biopsychosocial Religion and Health Study (BRHS) follow-up questionnaire.

**Table 4.** Relation of Vigorous-Activity Frequency to Waking Early and Not Going Back to Sleep, Adjusted for Control Variables (N = 3960).

Effect	Odds ratio	95% Cl^a^	*P*
Vigorous-activity frequency	1.02	(0.99, 1.06)	.141
Age	1.01	(1.01, 1.02)	**<.001**
BMI (Kg/m^2^)	1.00	(0.99, 1.02)	.647
Diet			
Omnivore	Ref.	​	​
vegan/lacto-ovo veg^b^	1.20	(1.04, 1.39	​
Education			
Less than high school	Ref.	​	​
Some college/AS/bachelors	0.78	(0.63, 0.96)	**.019**
Master’s degree or higher	0.86	(0.73, 1.03)	.099
Alcohol			
No	Ref.	​	​
Yes	0.86	(0.67, 1.11)	.239
Caffeinated-beverage use			
No	Ref.	​	​
Yes	0.90	(0.77, 1.05)	.172
Gender/race			
White male	Ref.	​	​
Black female	0.71	(0.58, 0.87)	**.001**
Black male	0.40	(0.31, 0.52)	**<.001**
White female	0.93	(0.79, 1.10)	.395
Weekend napping			
None	Ref.	​	​
<40 minutes	1.28	(1.03, 1.57)	**.023**
≥40 minutes	1.22	(1.03, 1.44)	**.021**
Weekday napping			
None	Ref.	​	​
<20 minutes	1.29	(1.08, 1.54)	**.006**
≥20 minutes	1.24	(1.02, 1.50)	**.031**
Interval between AHS-2 and BRHS^c^	1.04	(0.98, 1.10)	.229
AHS-2 - hours of sleeping			
6-8 hrs	Ref.	​	​
Less than 6 hrs	0.44	(0.33, 0.60)	**<.001**
9 hrs. or more	2.92	(2.30, 3.70)	**<.001**

*Note.* The dependent variable, waking early and not returning to sleep, was coded as 1 = yes (reported waking early and being unable to return to sleep) and 0 = no (did not report waking early or inability to return to sleep). Odds ratios greater than 1 indicate higher odds of waking early and not returning to sleep, whereas odds ratios less than 1 indicate lower odds, after adjustment for all covariates shown.

^a^CI = confidence intervals.

^b^Lacto-ovo vegetarian refers to a diet that excludes meat and fish but includes dairy products and eggs.

^c^Interval between AHS-2 and BRHS refers to the number of years between completion of the Adventist Health Study-2 (AHS-2) baseline questionnaire and the Biopsychosocial Religion and Health Study (BRHS) follow-up questionnaire.

In the multivariable logistic analysis shown in Table 2, after adjusting for all control variables, we found that the frequency of vigorous activity moderately protected against difficulty falling asleep (OR = 0.97; 95% CI = 0.94-0.98; *P* = .034).

In the multivariable analysis shown in Table 3, after adjusting for all control variables, we found that the frequency of vigorous activity did not protect against waking in the middle of the night (OR = 1.00; 95% CI = 0.97-1.03; *P* = .824).

In the multivariable logistic analysis shown in Table 4, after adjusting for all control variables, we found that the frequency of vigorous activity was not protective against waking early and being unable to go back to sleep (OR = 1.02; 95% CI = 0.99-1.06; *P* = .141).

Among the control variables, older age, weekday napping, and sleeping 9 hours or more per night were consistently associated with one or more adverse sleep outcomes.

## Discussion

We found that the frequency of vigorous activity might have been protective against difficulty falling asleep in a multiracial population. This relationship was small, but statistically significant after controlling for sociodemographic and lifestyle characteristics, beverage intake including alcohol and caffeine, as well as napping and total hours of sleep. Vigorous activity was not protective against waking in the middle of the night or waking early and being unable to fall asleep again. We did not have information regarding the timing of exercise in relation to sleep onset. Vigorous exercise close to bedtime is thought to disturb sleep.^15^ While this may be true, other researchers countered this notion, claiming that exercise-induced heat load from physical activity close to bedtime has little influence on sleep.^13^

A number of studies have previously shown an association between physical activity and sleep patterns. One of the first studies to link these two lifestyle determinants was the Alameda County [California] Health and Ways of Living Study started in 1965.^25^ The research found that exercise at least 5 days/week was related to increased sleep length. Prospective studies confirmed this relationship. Schroeder and colleagues found that regular physical activity, not weekend-only sessions, was inversely associated with inadequate sleep.^26^ Frequency of activity or walking a certain distance, regardless of intensity, was related to improved sleep. Moreover, as little as one instance of physical activity per week was inversely associated with waking up in the middle of the night. King, et al, showed in a randomized controlled clinical trial conducted in a mostly Caucasian population that four training sessions of physical activity per week shortened sleep latency and improved sleep duration.^27^ A recent systematic review found six studies conducted mostly in middle-aged populations. In this review, moderate aerobic and high-intensity resistance activities were combined.^28^ Physical activity was found to improve sleep quality and reduce latency, but did not increase duration or reduce sleep disturbance. Our findings also show that high-intensity vigorous aerobic physical activity, isolated from resistance activities, may have improved sleep latency, and similar to the review, we did not find an association with other sleep disturbances. However, these findings cannot assume causality.

In our study, compared to White males, Black women may have had greater difficulty falling asleep, but this gender and race characteristic may have protected against early awakening and being unable to get back to sleep. Black males had less difficulty falling asleep and were less likely to wake in the middle of the night or wake early and be unable to get back to sleep. Caucasian women had greater difficulty falling asleep and were more likely to wake in the middle of the night. In line with these findings, a recent review of prospective and case-control studies found that women are more susceptible to sleeping disorders than men.^29^

Our population was mainly a middle-aged to elderly age group in which sleep problems began to manifest themselves. Sleep problems typically increase with age and contribute to mortality risk in the elderly.^30^ In line with this finding, sleep difficulties were common in the study population, with prevalence of short sleep duration (<6 hours), difficulty falling asleep, waking in the middle of the night, and early awakening noted in about 9.3%, 36.1%, 48.96% and 44.2% of the participants, respectively. All four categories of sleep difficulties may be associated with increasing age.

Three sleep problems, namely difficulty falling asleep, waking in the middle of the night, and waking early and being unable to go back to sleep, may have been associated with short sleep duration. Our measure of sleep is virtually the same one used in previous studies,^31^ though not validated in the present study population. Our measure of physical activity was the Paffenbarger 1978, vigorous activity frequency question,^32^ “How many times per week do you usually engage in regular vigorous activities, long enough or with enough intensity to work up a sweat, get your heart thumping or get out of breath ?” High frequency of vigorous activity has been related to reductions in cardiovascular disease,^33^ but to our knowledge, this specific question has not been linked to sleep in previous studies. These data indicate that activity vigorous enough to protect against cardiovascular disease may also protect against long sleep latency.

This follow-up period was short and may be closer to a cross-sectional study. Exercise levels are probably stable in older adults, particularly among those with generally good health.^34^ Because we excluded respondents with chronic diseases, the factor that provides the biggest barrier to exercise stability in the elderly,^35^ a Spearman’s rho for our dataset was analyzed for exercise frequency, yielding a correlation of 0.51, which leads to an adequate assumption that exercise adherence has been reasonably stable throughout our study.

Strengths of our research include a study population that belonged to a faith-based group, which limits practices that may disturb sleep patterns, such as smoking and drinking alcohol or caffeinated-beverages.^24^ In our study population, there were only 45 individuals who smoked, and 406 participants who reported drinking alcohol. In addition, every attempt was made to recruit Black men and women, which consisted of ∼38% of the total study population.

Among confounders, caffeine use, which has been shown to disrupt sleep patterns, was surprisingly not associated with difficulty falling asleep.^36^ We found that a vegan diet may have had a small but still significant association with waking early and being unable to go back to sleep. There is no apparent explanation for this finding, which may be due to chance. From our results, there is a possibility of interpreting that having some college education was protective against waking early and not going back to sleep. Stamatakis and colleagues suggested that, on average, sleep length is positively related to the amount of schooling.^24^ Although we found no association between alcohol and any of the studied sleep patterns, Schoenborn and Adams^37^ concluded that 20% of chronic alcohol drinkers (five or more drinks per day) were more likely to experience short sleep.

Another potential confounder includes stress. Psychosocial stress may be associated with less exercise and sleep disturbances.^9^ Evidence has shown that stress (which turns into distress) negatively affects sleeping patterns.^38^ In the absence of a direct measure of stress, we tested a measure of difficulty meeting expenses in the past year as a proxy for psychosocial stress.^39^ We also controlled for both weekday and weekend napping. Napping tends to impair nighttime sleep,^19^ and our study confirmed that, in general, napping might have been associated with a greater likelihood of all three sleep problems.

Unexpectedly, obesity was not related to sleep disturbance in our study. The mean BMI of our population was ∼26 kg/m^2^, only slightly lower than the U.S. population mean of ∼30.21 kg/m^2^,^40^ which could be due in part to a healthy volunteer effect and the exclusion of the morbidly obese (BMI >40 kg/m^2^). Gohil and Hannon found that short sleep duration is strongly and consistently associated with concurrent obesity.^41^ Papatriantafyllou and colleagues suggested that loss of sleep promotes weight gain instead of the other way around, but this relationship is most likely bidirectional.^6^

There are several limitations to our study. Baseline measures corresponding to the three BRHS sleep symptoms were unavailable, so we could not adjust for pre-existing difficulty falling asleep, nocturnal awakening, or early-morning awakening, and we could not exclude participants based on poor sleep quality at baseline. We were unable to control for depression at baseline as well. Depression may be associated with both lack of exercise^42^ and sleep disturbances, as the latter can either be a cause of, or a result of depression.^43^ Because of the short follow-up period, we assume that mental illness may have been stable in the population.^44^

Other weaknesses include the self-reported nature of the data. However, previous studies have shown that self-reporting is consistent with direct (actigraphic) monitoring.^45^ Also, we did not have information on sleep apnea at baseline, a potent measure of sleep wellness.^46^ Our lack of data on night-shift work could be seen as another limiting factor since, according to Silva and Costa, this is a significant issue that should be considered when studying health-related associations with sleeping patterns.^47^ Night-shift work is a common characteristic of people who nap longer.^38^ Workers on the night shift exhibit longer weekend sleeping patterns when compared to weekday times, as if trying to “catch up” on lost sleep during the weekday.

## Conclusion

Among Seventh Day Adventists without selected medical conditions (as specified in the exclusion criteria), vigorous physical activity was significantly associated with a lower likelihood of difficulty falling asleep, suggesting that frequent engagement in high-intensity exercise may contribute to improved sleep habits among adults as a potential health benefit (despite no reported association with waking in the middle of the night or early-morning awakenings followed by difficulty returning to sleep). However, causal inference could not be established due to the observational design of the study. Additional research based on controlled interventions is therefore warranted to determine whether increasing vigorous activity directly improves sleep quality.

## Data Availability

AHS-2 and BRHS data used in this analysis are not publicly available for unrestricted download. Access to deidentified data may be considered for investigators after review and approval by the Adventist Health Study research team and completion of applicable institutional, ethical, and data-use requirements.*

## References

[1] Rottapel RE Zhou ES Spadola CE , et al. Adapting sleep hygiene for community interventions: a qualitative investigation of sleep hygiene behaviors among racially/ethnically diverse, low-income adults. Sleep Health. 2020;6(2):205-213. doi:10.1016/j.sleh.2019.12.009. 31983611 PMC7176530

[2] Lococo KH Staplin L Schultz MW . The effects of medical conditions on driving performance: A literature review and synthesis. National Highway Traffic Safety Administration. Accessed July 14, 2026. https://www.nhtsa.gov/sites/nhtsa.gov/files/documents/13394-mediconlitreview-073018-v3-tag.pdf

[3] Blackwelder A Hoskins M Huber L . Effect of inadequate sleep on frequent mental distress. Prev Chronic Dis. 2021;18:E61. doi:10.5888/pcd18.200573. Published 2021 Jun 17. 34138697 PMC8220958

[4] National Sleep Foundation . National Sleep Foundation Announces 2024 Dates for Sleep Awareness Week®. National Sleep Foundation. Accessed July 14 2026. https://www.thensf.org/dates-for-sleep-awareness-week-2024/

[5] Tomaso CC Johnson AB Nelson TD . The effect of sleep deprivation and restriction on mood, emotion, and emotion regulation: three meta-analyses in one. Sleep. 2021;44(6):zsaa289. doi:10.1093/sleep/zsaa289. 33367799 PMC8193556

[6] Papatriantafyllou E Efthymiou D Zoumbaneas E Popescu CA Vassilopoulou E . Sleep deprivation: effects on weight loss and weight loss maintenance. Nutrients. 2022;14(8):1549. doi:10.3390/nu14081549. 35458110 PMC9031614

[7] Xue H Zou Y Zhang S . The best modality and dose of physical activity to improve sleep quality in older adults: a Bayesian dose-response meta-analysis. Sleep Med. 2024;122:113-127. doi:10.1016/j.sleep.2024.08.008. 39154572

[8] Park I Díaz J Matsumoto S , et al. Exercise improves the quality of slow-wave sleep by increasing slow-wave stability. Sci Rep. 2021;11(1):4410. doi:10.1038/s41598-021-83817-6. Published 2021 Feb 24. 33627708 PMC7904822

[9] Alnawwar MA Alraddadi MI Algethmi RA Salem GA Salem MA Alharbi AA . The effect of physical activity on sleep quality and sleep disorder: a systematic review. Cureus. 2023;15(8):e43595. doi:10.7759/cureus.43595. Published 2023 Aug 16. 37719583 PMC10503965

[10] McCoy T Sochan AJ Spaeth AM . The relationship between sleep and physical activity by age, race, and gender. RCM (Rapid Commun Mass Spectrom). 2024;25(10):378. 10.31083/j.rcm2510378 PMC11522772 39484124

[11] Liu Y Wheaton AG Chapman DP Cunningham TJ Lu H Croft JB . Prevalence of healthy sleep duration among adults — united States, 2014. MMWR Morbidity and Mortality Weekly Report. 2016;65(6):137-141. doi:10.15585/mmwr.mm6506a1. 26890214

[12] Centers for Disease Control and Prevention . Physical Activity for Adults: An Overview. Centers for Disease Control and Prevention. Accessed July 14, 2026. https://www.cdc.gov/physical-activity-basics/guidelines/adults.html

[13] McGranahan MJ O'Connor PJ . Influence of regular physical activity on sleep. Curr Top Behav Neurosci. 2024;67:309-328. doi:10.1007/7854_2024_503. 39080238

[14] Tworoger SS Yasui Y Vitiello MV , et al. Effects of a yearlong moderate-intensity exercise and a stretching intervention on sleep quality in postmenopausal women. Sleep. 2003;26(7):830-836. doi:10.1093/sleep/26.7.830 14655916

[15] Tian D Meng J . Exercise for prevention and relief of cardiovascular disease: prognoses, mechanisms, and approaches. Oxid Med Cell Longev. 2019;2019:3756750. doi:10.1155/2019/3756750. Published 2019 Apr 9. 31093312 PMC6481017

[16] Sejbuk M Mirończuk-Chodakowska I Witkowska AM . Sleep quality: a narrative review on nutrition, stimulants, and physical activity as important factors. Nutrients. 2022;14(9):1912. doi:10.3390/nu14091912. Published 2022 May 2. 35565879 PMC9103473

[17] Weibel J Lin YS Landolt HP , et al. Regular caffeine intake delays REM sleep promotion and attenuates sleep quality in healthy men. J Biol Rhythms. 2021;36(4):384-394. doi:10.1177/07487304211013995. 34024173 PMC8276335

[18] Ortolá R Sotos-Prieto M García-Esquinas E Galán I Rodríguez-Artalejo F . Alcohol consumption patterns and mortality among older adults with health-related or socioeconomic risk factors. JAMA Netw Open. 2024;7(8):e2424495. doi:10.1001/jamanetworkopen.2024.24495. Published 2024 Aug 1. 39133491 PMC11320169

[19] Mograss M Abi-Jaoude J Frimpong E , et al. The effects of napping on night-time sleep in healthy young adults. J Sleep Res. 2022;31(5):e13578. doi:10.1111/jsr.13578. 35253300

[20] Butler TL Fraser GE Beeson WL , et al. Cohort profile: the adventist health Study-2 (AHS-2). Int J Epidemiol. 2007;37(2):260-265. doi:10.1093/ije/dym165. 17726038

[21] Tonstad S Butler T Yan R Fraser GE . Type of vegetarian diet, body weight, and prevalence of type 2 diabetes. Diabetes Care. 2009;32(5):791-796. doi:10.2337/dc08-1886. 19351712 PMC2671114

[22] Lee JW Morton KR Walters J , et al. Cohort profile: the biopsychosocial religion and health study (BRHS). Int J Epidemiol. 2009;38(6):1470-1478. doi:10.1093/ije/dyn244. 19052114 PMC2912533

[23] Singh PN Fraser GE Knutsen SF Lindsted KD Bennett HW . Validity of a physical activity questionnaire among African-American Seventh-day Adventists. Med Sci Sports Exerc. 2001;33(3):468-475. doi:10.1097/00005768-200103000-00021. 11252076

[24] Faul F Erdfelder E Lang AG Buchner A . G*Power 3: a flexible statistical power analysis program for the social, behavioral, and biomedical sciences. Behav Res Methods. 2007;39(2):175-191. doi:10.3758/bf03193146. 17695343

[25] Stamatakis KA Kaplan GA Roberts RE . Short sleep duration across income, education, and race/ethnic groups: population prevalence and growing disparities during 34 years of follow-up. Ann Epidemiol. 2007;17(12):948-955. 17855122 10.1016/j.annepidem.2007.07.096 PMC2140008

[26] Schroeder K Kubik MY Sirard JR Lee J Fulkerson JA . Sleep is inversely associated with sedentary time among youth with obesity. Am J Health Behav. 2020;44(6):756-764. doi:10.5993/AJHB.44.6.2. 33081874 PMC7890749

[27] King AC Oman RF Brassington GS Bliwise DL Haskell WL . Moderate-intensity exercise and self-rated quality of sleep in older adults. A randomized controlled trial. JAMA. 1997;277(1):32-37. 8980207

[28] Da Silva MAR Baptista LC Neves RS , et al. The effects of concurrent training combining both resistance exercise and high-intensity interval training or moderate-intensity continuous training on metabolic syndrome. Front Physiol. 2020;11:572. doi:10.3389/fphys.2020.00572. Published 2020 Jun 11. 32595518 PMC7300209

[29] Polo-Kantola P . Sleep problems in midlife and beyond. Maturitas. 2011;68(3):224-232. doi:10.1016/j.maturitas.2010.12.009. 21295422

[30] Tatineny P Shafi F Gohar A Bhat A . Sleep in the elderly. Mo Med. 2020;117(5):490-495. 33311760 PMC7723148

[31] Fabbri M Beracci A Martoni M Meneo D Tonetti L Natale V . Measuring subjective sleep quality: a review. Int J Environ Res Public Health. 2021;18(3):1082. doi:10.3390/ijerph18031082. Published 2021 Jan 26. 33530453 PMC7908437

[32] Paffenbarger RS Jr Wing AL Hyde RT . Physical activity as an index of heart attack risk in college alumni. American journal of epidemiology. 1978;108(3):161-175. doi:10.1093/oxfordjournals.aje.a112608. 707484

[33] Eijsvogels TM Molossi S Lee DC Emery MS Thompson PD . Exercise at the extremes: the amount of exercise to reduce cardiovascular events. J Am Coll Cardiol. 2016;67(3):316-329. doi:10.1016/j.jacc.2015.11.034. 26796398

[34] Izquierdo M Merchant RA Morley JE , et al. International exercise recommendations in older adults (ICFSR): Expert consensus guidelines. J Nutr Health Aging. 2021;25(7):824-853. doi:10.1007/s12603-021-1665-8. 34409961 PMC12369211

[35] Rhodes RE Martin AD Taunton JE Rhodes EC Donnelly M Elliot J . Factors associated with exercise adherence among older adults. An individual perspective. Sports Med. 1999;28(6):397-411. doi:10.2165/00007256-199928060-00003. 10623983

[36] Lieberman HR Agarwal S Caldwell JA Fulgoni VL . Demographics, sleep, and daily patterns of caffeine intake of shift workers in a nationally representative sample of the US adult population. Sleep. 2020;43(3):zsz240. doi:10.1093/sleep/zsz240. 31628471

[37] Schoenborn CA Adams PF . Sleep duration as a correlate of smoking, alcohol use, leisure-time physical inactivity, and obesity among adults: United States, 2004-2006. Centers for Disease Control and Prevention. Accessed July 14, 2026. https://www.cdc.gov/nchs/data/hestat/sleep04-06/sleep04-06.pdf

[38] Kalmbach DA Anderson JR Drake CL . The impact of stress on sleep: pathogenic sleep reactivity as a vulnerability to insomnia and circadian disorders. J Sleep Res. 2018;27(6):e12710. doi:10.1111/jsr.12710. 29797753 PMC7045300

[39] Patterson SL Sagui-Henson S Prather AA . Measures of psychosocial stress and stressful exposures. Arthritis Care Res. 2020;72(Suppl 10):676-685. doi:10.1002/acr.24228. PMC8063511 33091267

[40] Rader B Hazan R Brownstein JS . Changes in adult obesity trends in the US. JAMA Health Forum. 2024;5(12):e243685. doi:10.1001/jamahealthforum.2024.3685. 39671205 PMC11645646

[41] Gohil A Hannon TS . Poor sleep and obesity: concurrent epidemics in adolescent youth. Front Endocrinol. 2018;9:364. doi:10.3389/fendo.2018.00364. Published 2018 Jul 10. PMC6048236 30042730

[42] Pearce M Garcia L Abbas A , et al. Association between physical activity and risk of depression: a systematic review and meta-analysis. JAMA Psychiatry. 2022;79(6):550-559. doi:10.1001/jamapsychiatry.2022.0609. 35416941 PMC9008579

[43] Codella R Chirico A . Physical inactivity and depression: the gloomy dual with rising costs in a large-scale emergency. Int J Environ Res Public Health. 2023;20(2):1603. doi:10.3390/ijerph20021603. Published 2023 Jan 16. 36674363 PMC9862474

[44] Monteiro N Mari J Kieling C . A narrative historical review of psychiatric epidemiology in Brazil: focus on social and cultural determinants of mental health. Ssm - Mental Health. 2023;3(100212):100212. doi:10.1016/j.ssmmh.2023.100212.

[45] Robbins R Quan SF Barger LK , et al. Self-reported sleep duration and timing: a methodological review of event definitions, context, and timeframe of related questions. Sleep Epidemiol. 2021;1:100016. doi:10.1016/j.sleepe.2021.100016. 35761957 PMC9233860

[46] Malhotra A Ayappa I Ayas N , et al. Metrics of sleep apnea severity: beyond the apnea-hypopnea index. Sleep. 2021;44(7):zsab030. doi:10.1093/sleep/zsab030. 33693939 PMC8271129

[47] Silva I Costa D . Consequences of shift work and night work: a literature review. Healthcare (Basel). 2023;11(10):1410. doi:10.3390/healthcare11101410. Published 2023 May 12. 37239693 PMC10218650

